# The Efficacy of Conbercept in Polypoidal Choroidal Vasculopathy: A Systematic Review

**DOI:** 10.1155/2020/4924053

**Published:** 2020-08-13

**Authors:** Yimin Wang, Mengxi Shen, Jinwei Cheng, Xiaodong Sun, Peter K. Kaiser

**Affiliations:** ^1^Department of Ophthalmology, Shanghai General Hospital, Shanghai Jiao Tong University School of Medicine, Shanghai, China; ^2^Shanghai Key Laboratory of Fundus Diseases, Shanghai, China; ^3^Department of Ophthalmology, Bascom Palmer Eye Institute, University of Miami Miller School of Medicine, Miami, Florida, USA; ^4^Shanghai Engineering Center for Visual Science and Photomedicine, Shanghai, China; ^5^National Clinical Research Center for Ophthalmic Diseases, China; ^6^Cole Eye Institute, Cleveland, OH, USA

## Abstract

**Methods:**

Thirty studies with 1308 eyes were identified and included in this study. The primary outcome measures were best-corrected visual acuity (BCVA), and secondary outcomes were optical coherence tomography characteristics and polyp regression rates. The pooled results were calculated by the random-effect or fixed-effect model according to the heterogeneity of the data.

**Results:**

Despite a large standard deviation in means (SMD) improvement for BCVA and central retinal thickness (CRT) in the conbercept group, there was no statistically significant difference in the other outcomes compared to ranibizumab and aflibercept. However, there was a greater polyp regression rate in the conbercept group at 12 months.

**Conclusions:**

This systematic review indicates that conbercept may achieve similar BCVA and CRT improvements as ranibizumab and aflibercept, with a superior rate of polyp regression at 12 months.

## 1. Introduction

Polypoidal choroidal vasculopathy (PCV) is a subtype of neovascular age-related macular degeneration (nAMD) defined by orange-red bulb-like lesions and characteristic polypoidal features with or without a branching vascular network (BVN) on imaging [1, 2]. It was first described as posterior uveal bleeding syndrome [3] later described by Yannuzzi et al. as a choroidal vasculopathy which led to hemorrhage and exudation in 1990 [4]. It differs from typical choroidal neovascularization (CNV) in nAMD by the presence of serous or hemorrhagic pigment epithelial detachment (PED) which can cause serous retinal detachment or submacular hemorrhage leading to severe visual deterioration [5].

Antivascular endothelial growth factor (VEGF) therapy, with or without verteporfin photodynamic therapy (vPDT), has been widely used in PCV since randomized clinical trials demonstrated excellent efficacy [6, 7]. Several studies have shown that anti-VEGF monotherapy can stabilize the vision and decrease the exudation in PCV, but it had a limited impact on the polypoidal lesions, especially when using ranibizumab [8]. However, it is critical and common to use the polyp regression rate as a parameter in the studies to evaluate the outcome of PCV [7, 9].

The anti-VEGF medicines being used now are ranibizumab, bevacizumab, aflibercept, and conbercept. Ranibizumab is a humanized, affinity-matured, Fab fragment against all isoforms of VEGF-A that has been widely used in pivotal clinical trials and has solid evidence indicating improved best-corrected visual acuity (BCVA) and good safety in the management of PCV [10]. Bevacizumab is humanized monoclonal antibody against all isoforms of VEGF-A that has less clinical trial data but has been used successfully in PCV. Aflibercept and conbercept are recombinant fusion proteins composed of key domains from VEGF receptors 1 and 2 and are the only agents that bind all isoforms of VEGF-A, VEGF-B, and placental growth factor. The difference in the molecular structure is that conbercept has an additional domain 4 from VEGF receptor 2 which improves its binding affinity, isoelectric point, and half-life. Conbercept is currently FDA approved in China and undergoing worldwide phase 3 studies in nAMD [11], it has been proved to be safe and effective in nAMD in Chinese clinical trials [12] and has a lower price compared to the other approved anti-VEGF medicines in China.

Some meta-analyses have compared treatment options of PCV [13–15]. However, none of them have included conbercept. As a promising new treatment of PCV, it is important to evaluate the efficacy of conbercept in PCV, which will improve our understanding in the treatment options in PCV and optimize the management for PCV patients.

## 2. Materials and Methods

### 2.1. Literature Search

**Figure 1:**
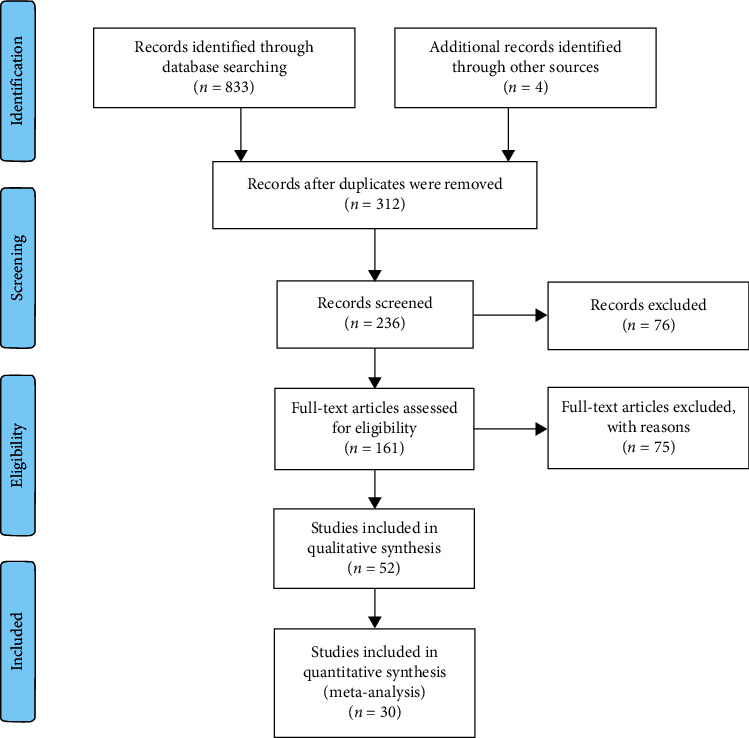
The selection flowchart of the studies included.

A PubMed, EMBASE, Clinical Trial, Web of Science, Cochrane Library, and Wanfang Database search using the search terms “polypoidal choroidal vasculopathy” or “PCV” and “vascular endothelial growth factor,” “anti-VEGF,” “ranibizumab,” “Lucentis,” “aflibercept,” “Elyea,” “conbercept,” or “Lumitin” was performed. The search did not have a limitation of language. All articles were downloaded for evaluation (Figure 1).

### 2.2. Inclusion and Exclusion Criteria

Studies were included if they met the following criteria: (1) there were clinical studies on human beings, (2) treatment included monotherapy with conbercept, ranibizumab, or aflibercept for the management of PCV, with or without placebo/control treatment, (3) proportion of VA improvements (visual gain more than 15 letters), the changes of BCVA, and central retinal thickness (CRT) as well as their standard deviation were available, and (4) polyp regression rates were available. Articles without full text, meeting reports, case reports, and repeat publications, were excluded from the analysis.

### 2.3. Data Extraction

The data was extracted in accordance with the following criterion: (1) the title, corresponding author, year of publication, the number of eyes, average age of patients, follow-up visits, gender composition, interventions, and retreatment regimen, (2) the mean BCVA and its standard deviation (SD) at baseline, (3) the mean BCVA and its standard deviation at month 12, (4) the mean CRT and SD at baseline, (5) the mean CRT and SD at month 12, (6) the percentage of polyp regression at month 12, (7) the mean injection numbers and SD at 12 months, and (8) the frequency of adverse effects, including subretinal hemorrhage and vitreous hemorrhage, which were recorded.

### 2.4. Outcome Measures

BCVA is a good indicator of retinal function. All visual acuity data were recorded and transformed into logMAR notation for statistical analysis. The CRT thickness was measured by ocular coherence tomography (OCT). The complete polyp regression rate was identified by ICGA. Month 12 was chosen as the time points for evaluating the effects of anti-VEGF treatment in PCV.

### 2.5. Quality Assessment

According to the Cochrane Handbook for Systematic Reviews of Interventions, we use GRADEpro software (GRADEproGDT 2015) to create the table of summary of find (SOF) (Table 1). BCVA and CRT at 12 months were included, as well as the polyp regression rate at 12 months. The quality of the evidence was judged using five aspects including limitation in study design, inconsistency, indirectness, imprecision, and reporting bias. The reasons why the quality of the studies was up- or downgraded are listed in the footnotes.

### 2.6. Statistical Analysis

Extracted data were analyzed by Comprehensive Meta-Analysis version 2 software. Standard deviation in means (SMD) with a 95% confidence interval was used for continuous variables, standard error (SEM), and variance were also calculated. We used fixed-effects model when there is no statistical heterogeneity (*I*^2^ > 50% or *p* < 0.1); otherwise, the random-effects model was selected. The significance of two anti-VEGFs was judged by *p* value. A *p* value <0.05 was considered significant, while *p* values >0.05 meant the two drugs performed equally.

## 3. Results

### 3.1. Search Results

A total of 833 articles were displayed after searching the keywords (Figure 1). After applying the inclusion and exclusion criteria, 30 studies with 1308 eyes were identified and included in the analysis. There are 4 arms for 0.5 mg/2.0 mg conbercept, 12 arms for 0.5 mg/2.0 mg ranibizumab, and 14 arms for 2.0 mg aflibercept. In studies comparing anti-VEGF monotherapy and PDT monotherapy, only data related to anti-VEGF monotherapy was included in our study.

### 3.2. Assessment of Evidence Quality

We used the “Grades of Recommendations Assessment, Development and Evaluation” (GRADE) system for evaluating the quality of evidence. The GRADE system would upgrade or downgrade the recommendation intensity according to several standards. As shown in Table 1, the GRADE of comparison of polyp regression rate between conbercept and ranibizumab at 12 months was low, while the others were very low.

Egger's measures were conducted to measure the asymmetry. The *p* value of Egger's measure of two outcomes “polyp regression rates” and “change in BCVA,” are higher than 0.05 (0.456 in polyp regression rates and 0.226 in changes of BCVA). However, p-Egger's of “change in CRT” *s* is 0.01.

### 3.3. Study Characteristics

The baseline characteristics of the analysis are shown in Table 2. The duration of the studies varied from 1 month to 36 months. All the studies included the BCVA improvement and CRT change. The rates of polyp regression identified by ICGA were contained in the studies.

### 3.4. Primary Outcome: Change in BCVA

According to the pooled-analysis of BCVA (Table 1), mean change in vision from baseline to 12 months all significantly improved in the conbercept group (*p*=0.004, 95% confidence interval (CI): 0.097 to 0.513), ranibizumab group (*p* < 0.0001, 95% CI: 0.127 to 0.421), and aflibercept group (*p* < 0.0001, 95% CI: 0.380 to 0.966). There was no significant difference when comparing any two of anti-VEGF agents (Table 3). The *p* value was 0.171 (95% CI: 0.352 to 0.797) and 0.811 (95% CI: 0.166 to 0.406) when comparing conbercept to aflibercept and ranibizumab, respectively. There's also no significant difference between aflibercept and ranibizumab (*p*=0.056, 95% CI: 0.327 to 0.693).

### 3.5. Secondary Outcome: The Polyp Regression Rates

The rate of polyp regression is shown in Tables 3 and 4. Conbercept showed a significantly greater polyp regression rate (event rate = 0.683) than ranibizumab (event rate = 0.324) at month 12 (*p* < 0.0001, overall 95% CI: 0.372 to 0.501). Conbercept has a better performance in polyp regression over aflibercept (event rate = 0.496) as well (*p*=0.032, 95% CI: 0.467 to 0.613). Though inferior to conbercept, aflibercept is superior to ranibizumab in terms of polyp regression (*p*=0.002, 95% CI: 0.367 to 0.489).

### 3.6. Secondary Outcome: Change in CRT

The pooled-analysis of CRT results is shown in Table 4. At month 12, conbercept (*p* < 0.0001, 95% CI: 0.690 to 1.476), ranibizumab (*p* < 0.0001, 95% CI: 0.688 to 0.950), and aflibercept (*p* < 0.0001, 95% CI: 1.098 to 1.772) showed a significant decrease in CRT based on OCT imaging. Similar to the change in BCVA, there was no significant difference between conbercept and the other two anti-VEGF medicines in terms of CRT (aflibercept: *p*=0.331, 95% CI: 1.063 to 1.610) (ranibizumab: *p*=0.144, 95% CI: 0.722 to 1.114). Interestingly, there is a significant improvement in CRT in the aflibercept group than the ranibizumab group (*p*=0.032, 95% CI: 0.976 to 1.473) (Table 3).

### 3.7. Secondary Outcome: Injection Frequency and Adverse Events

We conducted the pairwise comparison of injection frequency among three medicines at month 12 (Table 3). Aflibercept required more intravitreal injections than conbercept (*p*=0.01, 95% CI: 5.790 to 6.752) and ranibizumab (*p* < 0.0001, 95% CI: 5.719 to 6.523), while conbercept had the similar injection frequency as ranibizumab (*p*=0.501, 95% CI: 4.263 to 6.772). There was no difference in the adverse event rates among conbercept, ranibizumab, and aflibercept.

## 4. Discussion

PCV is a subtype of nAMD with characteristic clinical and imaging features that differentiate it from typical nAMD. It has been reported that the levels of VEGF are lower in the PCV eyes than typical nAMD eyes [45], possibly indicating a different underlying pathophysiology as well as treatment response to anti-VEGF therapy. Studies such as the PEARL study [46] (PCV management with different concentrations of intravitreal ranibizumab (IVR) monotherapy) and the LAPTOP study [47] (comparison of PDT versus IVR in PCV) have shown excellent efficacy of anti-VEGF agents in PCV. More recently, the EVEREST 2 and PLANET studies verified that anti-VEGF monotherapy can effectively treat PCV [48–50].

There have been numerous comparative studies of different anti-VEGF agents in a head-to-head manner in typical nAMD, but to date, no comparison studies in PCV management have occurred [8]. As a newly developed anti-VEGF medicine, limited studies focused on the efficacy of conbercept in comparison to ranibizumab and aflibercept in PCV, despite that conbercept is superior to ranibizumab in improving visual outcome in typical nAMD patients [51].

We performed this systematic review of published clinical studies evaluating conbercept and other anti-VEGF agents in the management of PCV. It showed significant improvement in BCVA and reduction in CRT among the three anti-VEGF agents at 12 months. In addition, conbercept showed the significantly highest rate of polyp regression at 12 months and less injection frequency than aflibercept. The conbercept and ranibizumab group are comparable since all of them were using a 3 + PRN (additional injections were given as needed after 3 initial monthly dose) regimen. However, almost half of the studies included for the injection frequency analysis in the aflibercept group were using a regimen that received every 2 months after 3 initial monthly doses, and one study is treat-and-extend regimen, while the rest were using a 3 + PRN regimen. Therefore, we want to remind readers to interpret these results with caution. As a structurally similar anti-VEGF to conbercept, aflibercept monotherapy has also demonstrated ideal efficacy in both PLANET [50] and VAULT study [43]. However, conbercept possesses better binding affinity and half-life due to its additional domain 4 from VEGFR2, which may improve its efficacy at 12 months.

CRT, a frequently used indicator to describe anatomic outcomes in nAMD studies [52], can also be used to evaluate treatment response in PCV. All the anti-VEGF agents in our study demonstrated significant decreases in CRT at month 12. Aflibercept has a slight advantage over ranibizumab, while there is no difference was found between aflibercept and conbercept. These results may indicate that there is a potential advantage of fusion proteins over ranibizumab on decreasing exudation and polyps.

One of the common parameters in PCV is polyp regression. Polyp regression rate is a crucial factor to the visual outcome in PCV patients, since the exudation and rupture of polyps would cause the enlargement of PED as well as massive hemorrhage [9, 53]. Thus, we looked at polyp regression rates in this meta-analysis. Conbercept demonstrated a significantly higher rate of polyp regression (with a mean event rate of 0.683) than ranibizumab (an event rate of 0.324) and aflibercept (an event rate of 0.496) at 12 months. Aflibercept monotherapy was reported to have a 38.9% complete polypoidal regression rate at week 52 in the PLANET study [50].The polyp regression rate was 33.8% in EVERESTII (ranibizumab monotherapy) [49] and 56.5% in the subgroup analysis of the AURORA study (conbercept monotherapy) [16]. Combining our results, it seems that conbercept has an advantage over aflibercept and ranibizumab in the closure of polyps, though the methodological differences among studies must be considered.

A possible explanation for the higher polyp regression rate of conbercept may be related to its high binding affinity for VEGF-A. By combining the lg-like domain of VEGFR1 and VEGFR2, it has a higher binding affinity for VEGF-A_165_ and lower isoelectric point than ranibizumab [54, 55]. Moreover, by design, conbercept binds placental growth factor (PGF) and VEGF-B. This approach is similar to aflibercept, another anti-VEGF fusion protein, that also has reported a higher rate of polyp regression than ranibizumab and bevacizumab [33, 42]. Aflibercept has a similar structure to conbercept, but with a lower binding affinity for VEGF-A, VEGF-B, and PGF. Moreover, we noticed that aflibercept had a significant decrease in CRT compared to ranibizumab, while conbercept did not. It may be due to the small quantity of study numbers in conbercept group and brings the wide range of confidence interval. The PANDA study (NCT03630952 and NCT03577899) is an international trial comparing conbercept with aflibercept, which will provide better data for this comparison of decrease in CRT with anti-VEGF therapy.

There are some limitations in our study. First, although we searched as many websites and databases as possible in order to include all the present studies, we may still have missed some papers, resulting in the publication bias. Second, since there was only a small quantity of randomized controlled trials (RCTs) with conbercept, most of the studies in conbercept group were retrospective which may lead to the ascertainment bias. Thus, we had to compare conbercept, aflibercept, and ranibizumab indirectly. These biases were downgraded in the SOF table, and our results were regarded as low-quality in GRADE system. The current definition of low quality evidence is that the confidence in the effect estimate is limited, but not worthless. Very low quality evidence stands for less confidence in the effect estimate. Therefore, we want to remind readers to interpret the results with caution. Obviously, high-quality RCTs and additional detailed data would help eliminate these biases in the future.

To our knowledge, this is the first systematic assessment of the efficacy of conbercept in PCV, using ranibizumab and aflibercept as references. Our study demonstrated that conbercept can achieve similar BCVA and CRT outcomes as ranibizumab and aflibercept during the first 12 months of treatment. Moreover, conbercept was superior to ranibizumab and aflibercept in outcome of polyp regression at one year. The data from ongoing randomized clinical trials will provide more details comparing anti-VEGF response between ranibizumab, aflibercept, and conbercept for the management of PCV.

## Figures and Tables

**Table 1:** Summary of findings.

Bibliography					
*The efficacy of conbercept in polypoidal choroidal vasculopathy-conbercept vs aflibercept*					
Outcomes	Number of participants (studies) Follow-up	Quality of the evidence (GRADE)	Relative effect (95% CI)	Anticipated absolute effects	
				Risk with aflibercept	Risk difference with conbercept (95% CI)
BCVA 12 months, Conbercept versus aflibercept logMAR	674 (17 studies) 12 months	⊕⊝⊝⊝ VERY LOW^1, 2, 3^ due to inconsistency, indirectness, large effect, plausible confounding would change the effect		The mean BCVA 12 months, conbercept versus aflibercept in the control groups was 0.665	The mean bcva 12 months, conbercept versus aflibercept in the intervention groups was 0.358 standard deviations lower (0.352 to 0.797 higher)
CRT 12 months, conbercept versus aflibercept oct	722 (19 studies) 12 months	⊕⊝⊝⊝ VERY LOW^1, 2, 3^ due to inconsistency, indirectness, large effect, plausible confounding would change the effect		The mean CRT 12 months, conbercept versus aflibercept in the control groups was 1.411	The mean crt 12 months, conbercept versus aflibercept in the intervention groups was 0.323 standard deviations lower (1.063 to 1.610 higher)
				Study population	
Polyp regression rate 12 months, conbercept versus aflibercept ICGA	643 (14 studies) 12 months	⊕⊝⊝⊝ VERY LOW^1, 2, 3^ due to inconsistency, indirectness, large effect, plausible confounding would change the effect	0.186 1 (0.467 to 0.613)	476 pr12 per 1000	217 more pr12 per 1000 (from 184 more to 254 more)
				Moderate	

*The efficacy of conbercept in polypoidal choroidal vasculopathy-conbercept versus ranibizumab*					
Outcomes	Number of participants (studies) Follow-up	Quality of the evidence (GRADE)	Relative effect (95% CI)	Anticipated absolute effects	
				Risk with ranibizumab	Risk difference with conbercept (95% CI)
BCVA 12 months, conbercept versus ranibizumab logMAR	442 (13 studies) 12 months	⊕⊝⊝⊝ VERY LOW^1, 2, 3, 4^ due to inconsistency, indirectness, large effect, plausible confounding would change the effect		The mean BCVA 12 months, conbercept versus ranibizumab in the control groups was 0.275	The mean bcva 12 months, conbercept versus ranibizumab in the intervention groups was 0.03 standard deviations higher (0.166 to 0.406 higher)
CRT 12 months, conbercept versus ranibizumab oct	336 (9 studies) 12 months	⊕⊝⊝⊝ VERY LOW^1, 2, 3, 4^ due to inconsistency, indirectness, large effect, plausible confounding would change the effect		The mean CRT 12 months, conbercept versus ranibizumab in the control groups was 0.781	The mean crt 12 months, conbercept versus ranibizumab in the intervention groups was 0.294 standard deviations higher (0.722 to 1.114 higher)
				Study population	
Polyp regression rate 12 months, conbercept versus ranibizumab ICGA	482 (9 studies) 12 months	⊕⊕⊝⊝ LOW^1, 2, 3, 4^ due to inconsistency, indirectness, large effect, plausible confounding would change the effect	0.373 1 (0.372 to 0.501)	333 pr12 per 1000	383 more pr12 per 1000 (from 166 more to 209 more)^3^
				Moderate	

^*∗*^The basis for the assumed risk (e.g., the median control group risk across studies) is provided in footnotes. The corresponding risk (and its 95% confidence interval) is based on the assumed risk in the comparison group and the relative effect of the intervention (and its 95% CI). CI: confidence interval. GRADE working group grades of evidence. High quality: further research is very unlikely to change our confidence in the estimate of effect. Moderate quality: further research is likely to have an important impact on our confidence in the estimate of effect and may change the estimate. Low quality: further research is very likely to have an important impact on our confidence in the estimate of effect and is likely to change the estimate. Very low quality: we are very uncertain about the estimate. ^1^Heteorgeneity. ^2^Indirect comparation. ^3^No explanation was provided. ^4^Different dose in conbercept group.

**Table 2:** The characteristics of studies.

Trials (author, year)	Location	Design	Treatment groups (patients)	Age (y), mean ± SD	Gender M/F	Interventions	Follow-up (month)
Qu et al., 2016 [16]	China	Retrospective subgroup analysis from RCT	0.5 mg IVC (*n* = 32) 2.0 mg IVC (*n* = 21)	66.5 ± 8.7 63.9 ± 9.6	12/20 8/13	0.5 mg conbercept 3 + PRN 2.0 mg conbercept 3 + PRN	3, 12
Qi et al., 2019 [17]	China	Prospective	IVC (*n* = 56)	68.21	31/25	0.5 mg conbercept 3 + PRN	3, 12
Cheng et al., 2016 [18]	China	RETRO	type1 PCV (*n* = 35) type2 PCV (*n* = 23)	66.5 ± 8.7 63.9 ± 9.6	22/13 13/10	0.5 mg conbercept 3 + PRN	3, 6, 9, 12
Liu et al., 2018 [19]	China	RETRO	IVC (*n* = 50)	66.58 ± 4.0	34/16	0.5 mg conbercept PRN	3, 6, 12
Oishi et al., 2013 [20]	Japan	RCT	IVR (*n* = 46) PDT (*n* = 45)	75.4 ± 6.9. 75 ± 8.0	28/18 32/15	0.5 mg ranibizumab 3 + PRN PDT 1 + PRN	12, 24
Lai et al., 2018 [21]	China	RCT	IVR (*n* = 18) PDT (*n* = 23) PDT + IVR (*n* = 16)	64.67 ± 8.52 60.52 ± 7.77 61.06 ± 9.12	12/6 14/9 10/6	0.5 mg ranibizumab 1 + PRN PDT 1 + PRN IVR + PDT 1 + PRN	1, 3, 6, 9, 12
Marcus et al., 2015 [22]	USA	RCT	2.0 mgIVR (*n* = 15) 0.5 mgIVR (*n* = 5)	62.6 51.8	8/7 3/2	Ranibizumab 3 + prn	3, 6, 9, 12
Ogino et al., 2013 [23]	Japan	Prospective	IVR (*n* = 23).	74.4 ± 6.9	18/5	0.5 mg ranibizumab 3 + PRN	3, 12
Kokame et al., 2014 [24]	USA	Prospective	IVR (*n* = 13)	76.31	11/2	0.5 mg ranibizumab monthly	1, 3, 6, 9, 12
Hikichi et al., 2014 [25]	Japan	Prospective	IVR (*n* = 86)	77 ± 8	62/24	0.5 mg ranibizumab 3 + PRN	12
Koh et al., 2017 [7]	Japan Korea Singapore Hong Kong Taiwan Thailand	RCT	IVR (*n* = 168) PDT + IVR (*n* = 154)	68.2 ± 9 68.0 ± 8.5	116/38 109/59	0.5 mg ranibizumab 3 + PRN PDT 1 + PRN/0.5 mg ranibizumab 3 + PRN	3, 12
Lai et al., 2011 [26]	Hong Kong	RETRO	IVR (*n* = 7) IVR + PDT (*n* = 16) PDT (*n* = 12)	64.6 ± 7.9 71.3 ± 9.8 65.6 ± 11.0	4/3 8/8 10/2	0.5 mg ranibizumab 3 + PRN PDT 1 + PRN/0.5 mg ranibizumab 3 + PRN PDT 1 + PRN	3, 12
Rouvas et al., 2011 [27]	Greece	RETRO	IVR (*n* = 10) IVR + PDT (*n* = 9) PDT (*n* = 11)	66.5 64.67 62.9	4/6 4/5 5/6	0.5 mg ranibizumab 3 + PRN PDT 1 + PRN/0.5 mg ranibizumab 3 + PRN PDT 1 + PRN	3, 6, 9, 12
Inoue et al., 2013 [28]	Japan	RETRO	IVR (*n* = 33) PDT (*n* = 44)	73.2 ± 7.5 71.0 ± 7.8	58/42 68/32	0.5 mg ranibizumab 3 + PRN PDT 1 + PRN	3, 6, 12, 18, 24
Sakai et al., 2016 [29]	Japan	RETRO	IVR (*n* = 20) IVR + PDT (*n* = 25)	75.3 ± 8.1 72.6 ± 6.2	13/7 21/4	0.5 mg ranibizumab 3 + PRN PDT 1 + PRN/0.5 mg ranibizumab 3 + PRN	6, 12, 18, 24, 30, 36
Cho et al., 2016 [30]	Korea	RETRO	IVA (*n* = 38) IVR (*n* = 60)	65.37 ± 9.24 63.22 ± 10.44	26/12 41/19	2 mg aflibercept 3 + PRN 0.5 mg ranibizumab 3 + PRN	3, 6, 9, 12
Takayama et al., 2017 [31]	Japan	RETRO	IVA (*n* = 11) IVA + PDT (*n* = 12)	73.5 ± 4.9 72.9 ± 5.5	6/5 6/6	0.5 mg aflibercept 3 + PRN PDT 1 + PRN/0.5 mg aflibercept 3 + PRN	1, 2, 3, 6, 9, 12
Kikushima et al., 2017 [32]	Japan	RETRO	IVA (*n* = 33) IVA + PDT (*n* = 33)	72.7 ± 8.5 73.4 ± 8.3	25/8 22/11	2.0 mg aflibercept 3 + PRN PDT 1 + PRN/2.0 mg aflibercept 3 + PRN	3, 6, 9, 12
Hara et al., 2016 [33]	Japan	RETRO	IVA (*n* = 29)	74 ± 8	22/6	2.0 mg aflibercept 3 + PRN	1, 2, 3, 6, 12
Arakawa et al., 2017 [34]	Japan	Prospective	IVA (*n* = 22)	62.5 ± 8.8	15/7	2.0 mg aflibercept 3 + Q2m	6, 12
Maruyama-Inoue et al., 2018 [35]	Japan	RETRO	IVA 3 + PRN (*n* = 10) IVA 3 + Q2m (*n* = 23)	67.8 ± 9.3 71.4 ± 7.8	6/4 18/5	2.0 mg aflibercept 3 + PRN 2.0 mg aflibercept 3 + Q2m	4, 12, 24, 36
Inoue et al., 2016 [36]	Japan	Prospective	IVA 3 + PRN (*n* = 25) IVA 3 + Q2m (*n* = 17)	71.1 ± 10.6 71.7 ± 7.1	13/4 19/6	2.0 mg aflibercept 3 + PRN 2.0 mg aflibercept 3 + Q2m	4, 6, 12
Ogasawara et al., 2018 [37]	Japan	RETRO	PCV (*n* = 64) AMD (*n* = 45)	72.7 ± 7.5 78.0 ± 10.2	48/16 37/8	2.0 mg aflibercept 3 + Q2m	Every month until month 12
Yamamoto et al., 2015 [38]	Japan	RETRO	IVA (*n* = 90)	71.1 ± 7.3	68/19	2.0 mg aflibercept 3 + Q2m	Every month until month 12
Yoneda et al., 2019 [39]	Japan	RETRO	IVA (*n* = 20) IVR + PDT (*n* = 43)	69.3 ± 7.5 73.3 ± 7.6	15/5 32/11	2.0 mg aflibercept 3 + Q2m PDT 1 + PRN/0.5 mg ranibizumab 3 + PRN	3, 6, 12, 24
Oshima et al., 2017 [40]	Japan	Prospective	IVA (*n* = 50)	73.6 ± 7.7	36/14	2.0 mg aflibercept 3 + Q2m	6, 12
Hosokawa et al., 2017 [41]	Japan	RETRO	IVA (*n* = 37)	73.6 (55–89)	30/7	2.0 mg aflibercept T&E	3, 6, 9, 12
Morimoto et al., 2017 [42]	Japan	RETRO	IVA (*n* = 58)	72.4 ± 1.1	45/13	2.0 mg aflibercept T&E	3, 6, 9, 12, 15, 18, 21, 24
Lee et al., 2017 [43]	Korea	Prospective	IVA (*n* = 40)	67.0 (44–84)	27/13	2.0 mg aflibercept 3 + Q2m	1, 2, 3, 4, 6, 8, 10, 12
Farooq et al., 2017 [44]	USA	Prospective	IVA (*n* = 20)	68 (46–90)	11/9	2.0 mg aflibercept 3 + Q2m	6, 12

IVC: intravitreal conbercept; IVR: intravitreal ranibizumab; IVA: intravitreal aflibercept; PDT: photodynamic therapy; PRN: pro re nata; T&E: treat and extend therapy.

**Table 3:** The comparison between groups.

Outcome	Treatment comparison			*p* value
	A	B	95% CI	
*Polyp regression rate*	Conbercept	Aflibercept	0.467 to 0.613	0.032
	Conbercept	Ranibizumab	0.372 to 0.501	<0.0001
	Aflibercept	Ranibizumab	0.367 to 0.489	0.002

*BCVA*	Conbercept	Aflibercept	0.352 to 0.797	0.171
	Conbercept	Ranibizumab	0.166 to 0.406	0.811
	Aflibercept	Ranibizumab	0.327 to 0.693	0.056

*CRT*	Conbercept	Aflibercept	1.063 to 1.610	0.331
	Conbercept	Ranibizumab	0.722 to 1.114	0.144
	Aflibercept	Ranibizumab	0.976 to 1.473	0.032

*Injection frequency*	Conbercept	Aflibercept	5.790 to 6.752	0.01
	Conbercept	Ranibizumab	4.263 to 6.772	0.501
	Aflibercept	Ranibizumab	5.719 to 6.523	<0.0001

*Adverse events*	Conbercept	Aflibercept	0.016 to 0.068	0.987
	Conbercept	Ranibizumab	0.020 to 0.072	0.879
	Aflibercept	Ranibizumab	0.027 to 0.061	0.912

**Table 4:** Standard mean difference and event rates.

Group	Outcome	Standard mean difference/event rates		Number of studies
		Mean	95% CI	
*Conbercept*	Polyp regression rate	0.683	0.552 to 0.790	3
	BCVA	0.305	0.097 to 0.513	4
	CRT	1.083	0.690 to 1.476	4
	Injection frequency	5.533	4.789 to 6.276	4
	Adverse events	0.026	0.003 to 0.175	5

*Ranibizumab*	Polyp regression rate	0.324	0.276 to 0.376	6
	BCVA	0.274	0.127 to 0.421	9
	CRT	0.819	0.688 to 0.950	5
	Injection frequency	5.030	4.267 to 5.793	4
	Adverse events	0.040	0.023 to 0.068	12

*Aflibercept*	Polyp regression rate	0.496	0.411 to 0.582	11
	BCVA	0.673	0.380 to 0.966	13
	CRT	1.435	1.098 to 1.772	15
	Injection frequency	6.811	6.176 to 7.445	5
	Adverse events	0.042	0.022 to 0.077	10
